# Use of Ceftolozane/Tazobactam for the Treatment of Multidrug-Resistant *Pseudomonas aeruginosa* Pneumonia in a Pediatric Patient with Combined Immunodeficiency (CID): A Case Report from a Tertiary Hospital in Saudi Arabia

**DOI:** 10.3390/antibiotics8020067

**Published:** 2019-05-27

**Authors:** Ahmed Zikri, Kamal El Masri

**Affiliations:** Department of Pharmacy, King Fahad Specialist Hospital, Dammam 31444, Saudi Arabia; kamal.masri@kfsh.med.sa

**Keywords:** ceftolozane/tazobactam, Pseudomonas, resistance, pneumonia, immunodeficiency

## Abstract

Infections, with multidrug-resistant *Pseudomonas aeruginosa*, are a major concern in the pediatric intensive care unit, especially in immunocompromised patients. Some of these strains are resistant to all beta-lactams, including carbapenems, leaving very limited treatment options remaining. These options include aminoglycosides and colistin, both of which have poor pharmacokinetic profiles with significant toxicities. Newer beta-lactam/beta-lactamase inhibitor combinations offer additional novel options to treat such infections, given their good pharmacokinetic profiles and activity against multi-drug resistant strains. Ceftolozane/tazobactam is a novel cephalosporin/beta-lactamase inhibitor combination approved in 2014. The drug demonstrates good activity against multidrug-resistant *P. aeruginosa* strains, including those resistant to all other antibiotics. Ceftolozane/tazobactam is currently approved in adult patients 18 years and older only. There are very limited data on its pharmacokinetic profile and clinical utility in the pediatric population. We report the use of ceftolozane/tazobactam to successfully treat pneumonia caused by multidrug-resistant *P. aeruginosa* in a pediatric patient with combined immunodeficiency syndrome.

## 1. Case Report

This is a case of a 14-year-old female patient (23 kg) diagnosed with Purine Nucleoside Phosphorylase Deficiency (PNP) syndrome, a form of combined immunodeficiency syndrome (CID). She had a history of recurrent respiratory infections and recurrent septic shocks requiring a recent several-months-long hospitalization in the pediatric intensive care unit at another tertiary hospital and was discharged two weeks prior to admission to our hospital. During that hospitalization, a bronchoalveolar lavage (BAL) culture was positive for methicillin-resistant *Staphylococcus aureus* (MRSA), *Pseudomonas aeruginosa*, and *Aspergillus flavus*. She received multiple antimicrobials, including meropenem, colistin, and amphotericin B. This was in addition to her prophylactic regimen, which included posaconazole, valganciclovir, trimethoprim/sulfamethoxazole, inhaled tobramycin, and inhaled pentamidine, in addition to monthly intravenous immunoglobulin (IVIG). 

Two weeks after discharge from the other hospital, the patient presented to our emergency department with a history of high-grade fever (39 °C on admission) with chills, increased work of breathing, cough, decreased activity, and oral intake. Upon assessment in the emergency department, she was found to have severe respiratory distress, for which she was given non-invasive mechanical ventilation (BiPAP). A chest X-ray was done on admission and revealed a bilateral bibasilar consolidation, which was more prominent on the right side, with mild pleural effusion. She was diagnosed with bilateral bronchopneumonia. She was also found to have a septic shock and she received fluid resuscitation and inotropic support for a few hours. She was started on meropenem 900 mg IV q8h (40 mg/kg/dose), vancomycin 330 mg IV q6h (14 mg/kg/dose), amikacin 360 mg IV daily (15 mg/kg/day), liposomal amphotericin B 100 mg IV daily (4 mg/kg/day), in addition to her prophylactic antimicrobial regimen listed above. 

Over the next 48 h, the patient remained febrile, without improving, necessitating the addition of colistin 2,000,000 IU IV q12h (3 mg/kg/dose of colistin base) to her antimicrobial regimen. Oral valganciclovir was also changed to intravenous ganciclovir. A BAL culture was sent five days after admission and revealed multidrug-resistant *Pseudomonas aeruginosa*, which was resistant to all beta-lactams, intermediate to ciprofloxacin, amikacin, and gentamicin, and only susceptible to colistin (Table 1). Antibiotics susceptibility testing was performed using VITEK^®^ 2 (bioMerieux, Paris, France). Confirmation of colistin susceptibility was done using Microscan^®^ broth microdilution test (Beckman Coulter, California, USA). All susceptibility results are reported according to Clinical & Laboratory Standards Institute (CLSI) breakpoints. An echocardiogram was done on the same day and showed moderate pulmonary hypertension, for which patient was started on oral sildenafil. All blood and urine cultures were negative.

Despite all antibiotics received by the patient, her condition continued to deteriorate. She remained febrile, with each febrile episode lasting approximately four hours. When she had chills, she had increased oxygen requirement and looked very toxic. She also needed BiPAP continuously, with increased requirements to meet her rising PCO2. A Carba-R^®^ PCR test (Cepheid, CA, USA) was negative for the five main carbapenemases (OXA-48, KPC, VIM, IMP, and NDM). Susceptibility testing to ceftolozane/tazobactam and ceftazidime/avibactam was performed using E-test® (bioMerieux, Paris, France). Organism was susceptible to ceftolozane/tazobactam but resistant to ceftazidime/avibactam, so the decision was made to start her on ceftolozane/tazobactam. She was started on 1.5 grams IV every 8 h (44/22 mg of ceftolozane/tazobactam/kg/dose) on the sixth day of hospital admission. Meropenem, vancomycin and liposomal amphotericin B were stopped. 

Forty-eight hours after initiating ceftolozane/tazobactam, the patient started to show signs of improvement, with less persistent fever, improved activity, and decreased requirement for BiPAP. Over the next 24 h that followed, she was shifted from BiPAP to high flow nasal cannula (20 L/min). Four days after the initiation of ceftolozane/tazobactam, the patient was afebrile for the first time since admission. Six days after initiation of the novel agent, the patient significantly improved, was tolerating oxygen supplementation via nasal cannula (2 L/min) and was safely transferred to the general pediatric ward on ceftolozane/tazobactam, amikacin, and colistin. No adverse events related to the use of ceftolozane/tazobactam were reported during the entire course of hospitalization. A follow-up CT scan, performed three weeks after admission, showed bilateral consolidation, more prominently seen in the right middle lobe, as well as bronchiectasis, which was markedly seen in the right middle lobe, and in both lower lobes. Consent was obtained from patient’s father and the Institutional Review Board (IRB) approved the publication of this manuscript.

## 2. Discussion

Multidrug-resistant *P. aeruginosa* remains a global threat that affects adult and pediatric patients alike. The availability of very limited treatment options, such as aminoglycosides and colistin, and the poor pharmacokinetic profiles coupled with the toxicities of these agents make it a significantly more challenging problem. The recent approval of novel beta-lactam/beta-lactamase inhibitor combinations offers clinicians additional options for treating these difficult infections. However, these agents are only approved in adult patients and there are very limited data regarding their use in the pediatric population, and even less data on their use in critically ill children.

In this report, we describe the safe use of ceftolozane/tazobactam to successfully treat a critically ill child with CID presenting to our hospital with pneumonia caused by a multidrug-resistant *P. aeruginosa* strain, which was resistant to all beta-lactams while only fully susceptible to colistin. 

CID is a rare, potentially fatal syndrome of diverse genetic causes, which leads to a combined absence of T-lymphocyte and B-lymphocyte function. Severe infections are the most common presenting symptom of patients with CID. Infections are usually caused by opportunistic organisms that would not otherwise cause infections in healthy children [1]. The overuse of antibiotics and the rise of multidrug resistance in opportunistic pathogens such as *P. aeruginosa* create a major challenge when treating these patients. Multidrug-resistant strains are now resistant to most, if not all, beta-lactams, with very limited options remaining. The poor pharmacokinetic profile of the remaining options, such as aminoglycosides and colistin, coupled with the toxicity profile of these agents warrants the development of new agents that are safer with an expanded coverage against these resistant organisms. 

Ceftolozane/tazobactam is a novel cephalosporin/beta-lactamase inhibitor combination that is active against many gram-negative bacteria, including some multidrug-resistant *P. aeruginosa* strains. The drug is not active against carbapenemase-producing strains, but it has good activity against *P. aeruginosa* strains that are resistant to carbapenems via mechanisms other than carbapenemase production. One common mechanism is the loss of the outer membrane porin D (OprD) function, which is commonly seen in combination with other mechanisms, such as over-expression of AmpC beta-lactamases or overexpression of efflux pumps. Ceftolozane is an oxyimino-aminothiazolyl cephalosporin that demonstrates stability against chromosomal AmpC Beta-lactamases, overexpressed MexAB-OprM efflux pumps, and deleted OprD porins [2].

Ceftolozane/tazobactam is only approved in patients 18 years and older, and there is very limited data on its use in pediatric patients. A phase I single-dose pharmacokinetic study of ceftolozane/tazobactam in pediatric patients <18 years of age indicated that the drug was well tolerated in this population, with no adverse events reported. It also concluded that doses of 1000/500 mg in patients 12 to 18 years and 20/10 mg/kg in patients <12 years old produced ceftolozane/tazobactam exposure similar to those in adult patients [3].

There is also very limited data demonstrating clinical efficacy of ceftolozane/tazobactam in pediatric patients. Aitken and colleagues reported the safe and effective use of ceftolozane/tazobactam in treating a multidrug-resistant *P. aeruginosa* bloodstream infection in a 9-year-old boy with acute myeloid leukemia [4]. In another report, a 14-year old female, with a history of Cystic Fibrosis, who presented with respiratory infection, caused by multidrug-resistant *P. aeruginosa*, was successfully treated with ceftolozane/tazobactam after multiple agents including ciprofloxacin, amikacin, and piperacillin/tazobactam have failed [5]. Currently, there are two ongoing clinical trials evaluating the use of ceftolozane/tazobactam for treating complicated urinary tract infections (clinicalTrials.gov NCT03230838) and complicated intra-abdominal infections (clinicalTrials.gov NCT03217136) in pediatric patients.

In this case report, the use of ceftolozane/tazobactam was safe and effective in the treatment of multidrug-resistant *P. aeruginosa* infection in the lungs of a patient with CID. A negative Carba-R^®^ PCR test suggests that resistance is most likely not carbapenemase-mediated, and is probably due to OprD loss or downregulation, overexpression of AmpC beta-lactamases, overexpression of efflux pumps, or a combination of two or all of these mechanisms. Since our patient was immunocompromised and given the lack of experience with the use of ceftolozane/tazobactam in pediatric patients and its ability to achieve adequate concentrations in lung tissues, with the recommended dose of 1500 mg IV q8h, per the phase I single-dose pharmacokinetic study cited above, we decided to continue amikacin and colistin to decrease the risk of clinical failure on ceftolozane/tazobactam monotherapy. 

While the drug is only approved for the treatment of complicated intra-abdominal infections (in combination with metronidazole) and complicated urinary tract infections in adults, applications have been submitted to the FDA and the European Medicines Agency to expand its indications to be used for treatment of nosocomial pneumonia (hospital-acquired and ventilator-associated). In our case, the patient responded very well to treatment. She became afebrile within four days of initiating treatment, with marked improvement in her respiratory symptoms, and was safely discharged from the pediatric intensive care unit six days after the initiation of ceftolozane/tazobactam. 

Our report highlights the use of newer beta-lactam/beta-lactamase inhibitor combinations, such as ceftolozane/tazobactam in treating infections caused by multidrug-resistant strains, which are resistant to all other antibiotics, or by strains susceptible to some antibiotics with lack of clinical response, due to poor tissue penetration, as was the case for this patient. 

This report is another addition to the very limited data available in the literature regarding the use of ceftolozane/tazobactam in pediatric patients. Larger clinical trials are underway to further evaluate its safety and efficacy in this patient population.

## Figures and Tables

**Table 1:** Antimicrobial susceptibility results for multidrug-resistant *Pseudomonas aeruginosa* from bronchoalveolar lavage culture (Results are from VITEK^®^ 2 unless otherwise noted).

Antimicrobial	MIC	Interpretation	Antimicrobial	MIC	Interpretation
Amikacin	32	I	* Ertapenem		R
Gentamicin	8	I	Imipenem	≥16	R
* Cefalexin		R	Meropenem	≥16	R
* Cefuroxime		R	Piperacillin/tazobactam	≥128	R
Ceftazidime	≥64	R	** Colistin	≤2	S
Cefepime	≥64	R	*** Ceftazidime/avibactam	48	R
Ciprofloxacin	2	I	*** Ceftolozane/tazobactam	3	S

S = Susceptible, I = Intermediate, R = Resistant, * Deduced Drug, ** Broth microdilution test, *** E-test.
